# Monitoring SARS-CoV-2 IgA, IgM and IgG antibodies in dried blood and saliva samples using antibody proximity extension assays (AbPEA)

**DOI:** 10.1038/s41598-024-72453-5

**Published:** 2024-09-17

**Authors:** Mengqi Wang, Masood Kamali-Moghaddam, Liza Löf, Matilde Cortabarría Fernandez, Roger Díaz Codina, Fredrik H. Sterky, Mikael Åberg, Ulf Landegren, Hongxing Zhao

**Affiliations:** 1 Department of Immunology, Genetics and Pathology, Science for Life Laboratory, Uppsala University, Uppsala, Sweden; 2 Unit of Affinity Proteomics Uppsala, Science for Life Laboratory, Uppsala, Sweden; 3 Department of Medical Sciences, Uppsala University, Uppsala, Sweden; 4 Department of Laboratory Medicine, University of Gothenburg, Gothenburg, Sweden; 5 Wallenberg Centre for Molecular and Translational Medicine, University of Gothenburg, Gothenburg, Sweden; 6 Department of Clinical Chemistry, Sahlgrenska University Hospital, Gothenburg, Sweden; 7 Present Address: Institut Pasteur de Montevideo, Montevideo, Uruguay

**Keywords:** Immunoassays, Antibody proximity extension assay, Antibody isotypes, IgG, IgM, IgA, SARS-CoV-2 antibody, Vaccination, Dried blood spot (DBS), Dried saliva spot (DSS), Real-time PCR, Biotechnology, Immunology

## Abstract

Using a modified proximity extension assay, total and immunoglobulin (Ig) class-specific anti-SARS-CoV-2 antibodies were sensitively and conveniently detected directly from ø1.2 mm discs cut from dried blood and saliva spots (DBS and DSS) without the need for elution. For total Ig detection, antigen probes were prepared by conjugating recombinant spike protein subunit 1 (S1-RBD) to a pair of oligonucleotides. To detect isotype-specific antibody reactivity, one antigen probe was replaced with oligonucleotide-conjugated antibodies specific for antibody isotypes. Binding of pairs of oligonucleotide-conjugated probes to antibodies in patient samples brings oligonucleotides in proximity. An added DNA polymerase uses a transient hybridization between the oligonucleotides to prime synthesis of a DNA strand, which serves as a DNA amplicon that is quantified by real-time PCR. The S1-RBD-specific IgG, IgM, and IgA antibodies in DBS samples collected over the course of a first and second vaccination exhibited kinetics consistent with previous reports. Both DBS and DSS collected from 42 individuals in the autumn of 2023 showed significant level of total S1-RBD antibodies with a correlation of R = 0.70. However, levels in DSS were generally 10 to 100-fold lower than in DBS. Anti-S1-RBD IgG and IgA in DSS demonstrated a correlation of R = 0.6.

## Introduction

Assays for antibody response to infectious pathogens provide valuable insight in the immune status of individuals as well as population immunity levels and disease prevalence. Similarly, specific antibody responses are of diagnostic value in autoimmunity and reactivity to neoantigen, tumor-specific mutant proteins, can be of importance for cancer patients.

Enzyme-linked immunosorbent assay (ELISA) is the most commonly used method for antibody detection in clinical laboratories. In recent years there has been a rapid development of immunoassays, such as lateral flow assays (LFA), chemiluminescence immunoassays (CLIA), the Luminex assay, and Meso scale discovery (MSD). These assays differ in aspects such as convenience, sensitivity, assay duration, dynamic range and potential for multiplexing. LFA are suitable at the point-of-care, but sensitivity is limited. CLIA tests have excellent sensitivity, specificity and dynamic range. Luminex assays use a set of color-coded beads with distinct spectral signatures for multiplex analyses of up to 100 antibody specificities. All these methods use labeled antibody class-specific antibodies for detection of donor antibodies that have bound to antigen coupled on a solid support. For these solid-phase assays, samples spotted on paper such as dried blood spot (DBS) or dried saliva spot (DSS), first must be extracted from the paper samples.

Two recently described DNA-assisted assays of antibody reactivity: agglutination-PCR (ADAP)^1–3^ and antibody proximity extension assay (AbPEA)^4^ differ from traditional assays in several respects. These assays use pairs of oligonucleotide-conjugated antigens that are brought in proximity when single or multiple antigen molecules are brought in close by being bound via bi- or multivalent antibodies that may recognize multiple epitopes on the antigen molecules. The resulting clusters account for the high sensitivity of these assays. Amplifiable DNA strand are generated through ligation by a DNA ligase via hybridization to a third oligonucleotide (ADAP)^1,2^, or by the extension of two hybridized oligonucleotides via DNA polymerization (AbPEA)^4^. The amplicons then serve as surrogate markers for the antigen-specific antibodies, with readout through real-time PCR. No secondary antibodies are needed to measure total antibody responses in these assays. Therefore the same assays can be used in different animal species for detection of antibodies directed against a given pathogen. Since assays are performed in solution phase there are no washing steps. The assays lend themselves for parallel analyses by conjugating barcoded oligonucleotides to pairs of many different antigens. AbPEA has been shown to be suitable for detection of antigen-specific antibody responses using DBS^4^. The reagents can be added directly to punched-out portions of samples dried on paper and incubated without prior elution, followed by a dilution step with addition of a DNA polymerase to create an amplicon for detection. Our previous studies demonstrated an interassay CV of < 8.1% for AbPEA, and we showed that anti-SARS-CoV-2 antibodies could be detected by AbPEA 5 to 10 days after infection or a first vaccination^4^. We also compared our assay to more standard antibody assays.

Collection of DBS by finger pricks has proven advantageous for monitoring blood protein and antibody levels, as samples can be collected at home without the need for medical personal and sent by post for analysis. Previous studies have demonstrated that levels of antibodies against SARS-CoV-2 detected in DBS samples correlate well with those of samples obtained by venipuncture^4–6^. Nonetheless, fingertip blood collection may still be considered somewhat invasive and uncomfortable, particularly for children or with frequent testing. In this regard, saliva is an ideal specimen that has gained popularity in recent years, and antibodies in saliva represent both mucosal and systemic immunity^7–11^. DSS remain stable for long periods of time, simplifying transportation and storage^12^. Antibody levels in saliva are lower than in serum, but linear correlation has been reported in most studies^9,11,13,14^. In general, IgG in saliva showed a better correlation to blood levels than IgA. Significantly, IgA in saliva has proven responsible for neutralizing the SARS-CoV-2 virus, thus serving an important role in mucosal immunity^15^.

Different antibody isotypes display distinct kinetics in responses to SARS-CoV-2 infection^16,17^. It has been shown that both IgM and IgA levels rise within 3 weeks before declining, while IgG reach a peak between three and seven weeks after symptom onset and then maintain plateau levels for a long time^18^. Analysis of antibody isotypes can thus provide information about the course of infection or vaccination. In our previous studies, only total blood antibody levels were detected using AbPEA.

In this study, total anti-SARS-CoV-2 S1-RBD antibodies in DSS and DBS, collected from 42 individuals in the autumn of 2023, were investigated using AbPEA. In addition, we adapted AbPEA for detection of antigen-specific IgG, IgM and IgA. These isotype-specific assays were evaluated using DBS, serially collected throughout first and second vaccinations, previously analyzed for total antibody levels^4^. IgA and IgG level were also compared in DSS and DBS from 42 individuals.

## Results and discussion

Detection of isotype-specific antibodies responses to SARS-CoV-2 vaccination using DBS samples.

**Fig. 1:**
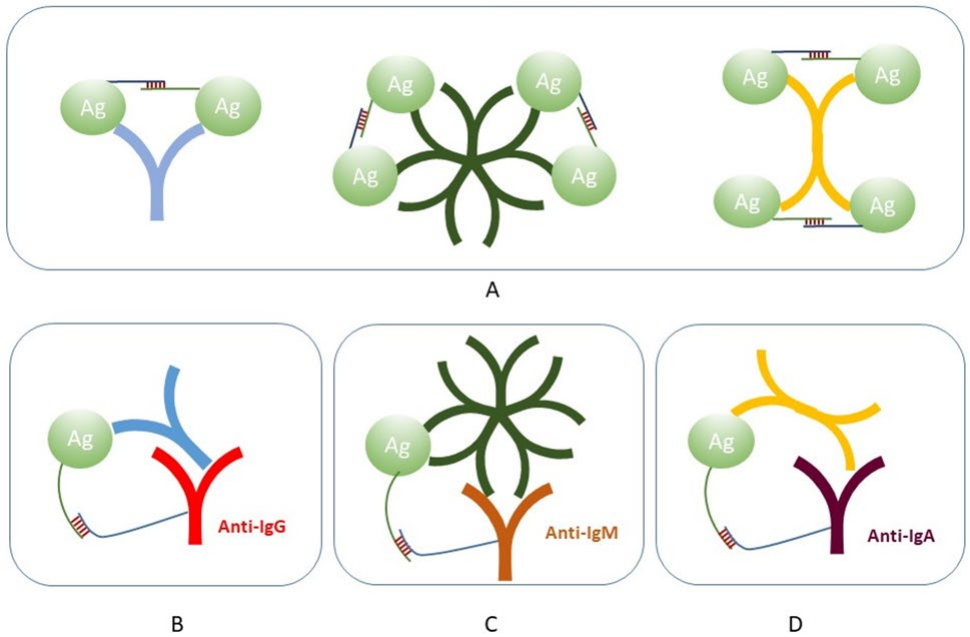
Schematic illustration of AbPEA for total and isotype specific antibody responses. (**A**) The previously described AbPEA with pairs of oligonucleotide-conjugated antigen molecules for measuring total antibody responses against a given antigen. Upon incubation with a patient sample, pairs of antigen probes bind antigen-specific antibodies in the patient sample, bringing their conjugated pairs of oligonucleotides in proximity. This allows an added DNA polymerase to extend the oligonucleotides to create amplifiable DNA strands, which contain barcodes from pairs of oligonucleotides conjugated to a particular antigen for total antibody detection. (**B–D**) AbPEA modified for detection of antigen-specific antibodies of isotypes IgG, IgM and IgA, respectively. One of the oligonucleotide-conjugated antigen molecules is replace by oligonucleotide-conjugated anti-human IgG, or IgA or IgM antibodies. The DNA extension products from simplex or multiplex reactions are then amplified using universal primers to generate sufficient amplicons. Finally, antigen-specific amplicons are measured by real-time PCR, recording the PCR cycle (Ct) at which the signal exceeds a threshold value. ΔCt values represent the difference between the Ct of the negative control and that of the sample.

In our previous study, we developed a novel homogeneous PCR-based assay, AbPEA, for sensitive and specific detection of SARS-CoV-2 antibodies in both serum and DBS after infection or vaccination^4^. In this assay, antigen probes were prepared by conjugating the recombinant spike protein subunit 1 containing the receptor binding domain (S1-RBD) of SARS-CoV-2, to each of a pair of specific oligonucleotides. Upon incubation with serum or DBS samples, the bi- or multivalency of the antibodies (IgG, IgA or IgM) brings pairs of viral proteins with their conjugated oligonucleotides in proximity (Fig. 1 panel A). This allows their attached oligonucleotides to hybridize and be extended by a DNA polymerase. The DNA extension products are then amplified and quantified by real-time PCR using specific primers and molecular beacons.

This antibody PEA (AbPEA) test uses only 1 µl of neat or up to 100,000-fold diluted serum or one ø1.2 mm disc cut from a DBS^4^. However, our early analysis focused solely on total antibodies, and different antibody isotypes could not be distinguished. The different antibody isotypes play separate roles, exhibit differential distribution in bodily fluids, and follow separate kinetics upon immunization, whether in response to natural infection or vaccination. To address this limitation, we modified AbPEA, as illustrated in Fig. 1B–D, enabling the detection of different isotypes of SARS-CoV-2-specific antibodies. In these variants of the assay, one of the oligonucleotide-conjugated antigen reagents was replaced by oligonucleotide-conjugated Ig class-specific antibodies against either human IgG, IgM or IgA. Different concentrations of oligonucleotide-conjugated antibody class-specific antibodies were tested. The best signal over background was obtained when the antibody conjugates were added at 20 nM in singleplex assays (data not shown).

Eleven DBS samples, serially collected from two individuals during the course of two vaccinations, were used to examine the kinetics of total antibodies, as well as IgM, IgA and IgG responses. Paper discs, 1.2 mm in diameter (corresponding to approximately 0.5 µl fresh blood), were cut from a DBS and either added directly into the incubation solution (before the second vaccination) or eluted and used as 1 µl of 100-fold (after second vaccination). Our previous report indicated that the recorded antibody levels in ø1.2 mm cuts from DBS collected after a second vaccination were at plateau levels. Therefore, a 100-fold dilution was necessary for accurate quantification using AbPEA. Following incubation for one hour at 37 °C or overnight at 4 °C, PCR buffer was added diluting the reactions, and then extension/prePCR and real-time PCR were performed. The results were presented by plotting ΔCt, which in log2 scale was calculated as the difference between the PCR cycle (Ct) of results for a sample collected before vaccination and the measured values at different time points after vaccination.

**Fig. 2:**
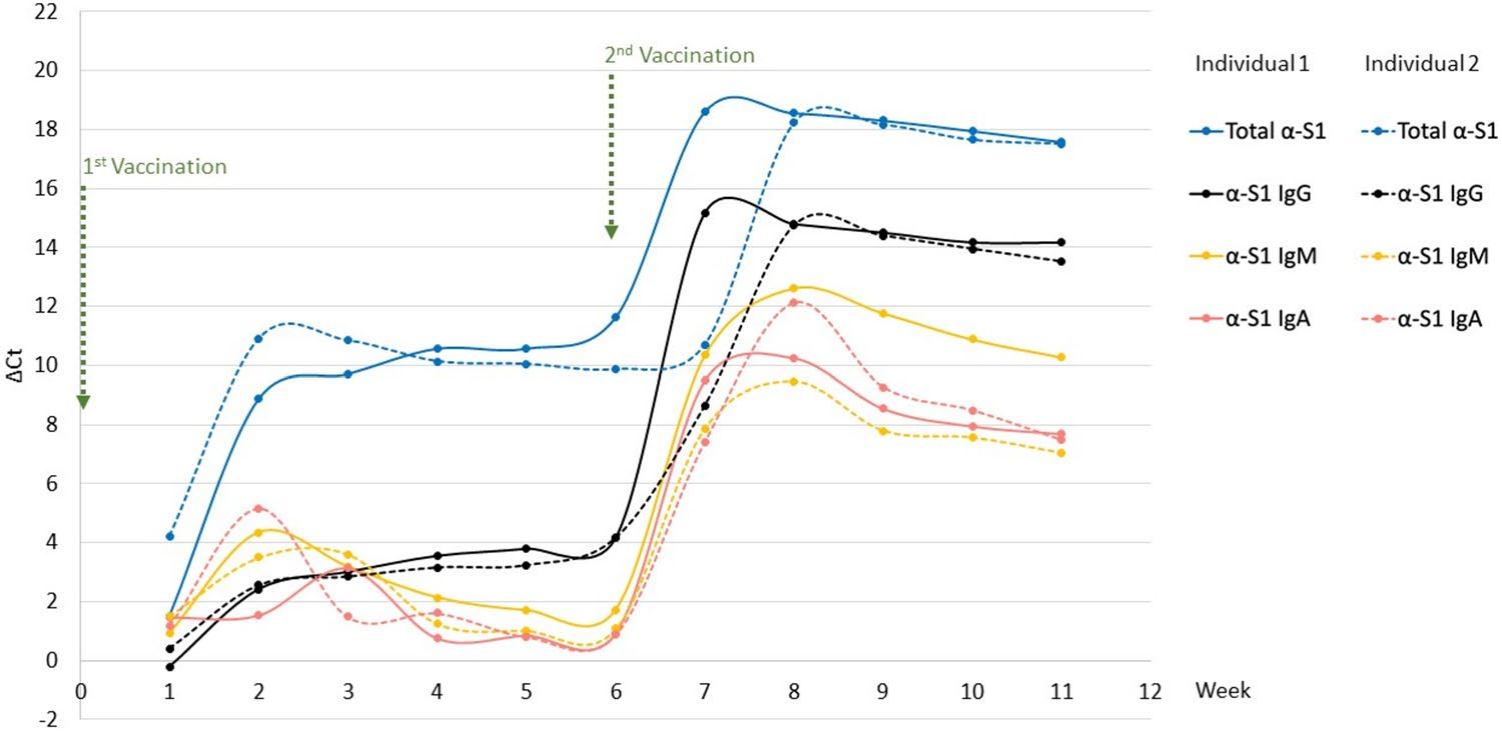
Kinetics of IgM, IgA and IgG responses by two individuals to vaccination against SARS-CoV-2. DBS samples were collected weekly after a first vaccination until 5 weeks after a second vaccination. In total eleven DBS samples from each of two individuals were examined. Paper discs, 1.2 mm in diameter (corresponding to approximately 0.5 µl fresh blood), were cut from DBS and either added directly into the incubation solution or eluted and used as 1 µl of a 100-fold dilution. Results for individual 1 are indicated by a solid line, and a dashed line is used for sample donor 2. The times of vaccinations are indicated by green arrows. ∆Ct was calculated by comparison to a negative control obtained before 2019. The antibody levels after second vaccination were measured after 100-fold dilution, but their ∆Ct values were calculated by adding 6.64 (the log2 100] to compensate for the 100-fold dilution. Total Ig, IgG, IgM and IgA were indicated by blue, black, yellow and red lines.

The results of total antibody detection accorded with those reported in our previous study^4^, increasing to a maximum at two weeks after a first vaccination, and sustained until a second vaccination further increased antibody levels (Fig. 2). Both IgA and IgM antibody responses displayed distinct kinetics compared to measurement of total antibody levels. IgA and IgM levels peaked between 2–3 weeks after each vaccination and then fell rapidly to low level during weeks 3–4. IgG reached the highest level around 4 weeks after a first vaccination, which then remained stable until a second vaccination further increased IgG levels. All three antibody isotypes showed a similar response to a second vaccination, peaking at 1–2 weeks. However, IgA and IgM remained at high levels for two weeks and then decreased gradually, while IgG remained relative stable up to the final week 11 tested here. The results did not permit comparison of the absolute levels of the different antibody isotypes, since the recorded levels also depended on factors such as the affinity of the isotype-specific antibodies used for detection. Nonetheless, the kinetics of total antibody levels displayed a similar profile as the sum of all antibody isotypes. The kinetics of IgM, IgA and IgG were well in line with the known kinetics of responses for the different antibody isotypes^16^.

### Detection of SARS-CoV-2 antibodies in DSS

Saliva has become increasingly important as an alternative to blood for detecting antibodies in infectious diseases, due to the non-invasive and convenient collection method. Dried saliva samples (DSS) offer significant advantages over liquid saliva due to easier transport, storage and maintain the stability of proteins and antibodies. Most available assays require an elution or extraction step, and in some cases a concentration step is also necessary. Detection of anti-S1-RBD antibody in DSS by AbPEA was evaluated here. DSS samples were collected by a simple procedure: saliva was deposited directly on filter papers. Alternatively, saliva was collected in a micro tube, and then 10–25 µl saliva was pipetted onto filter paper. Similar results were obtained for DSS collected with or without first rising the mouth with water (data not shown).

To compare antibody levels in DSS versus liquid saliva, both sample types were collected in parallel from five individuals. One cut of ø1.2 mm disc (equal to 0.25 µl liquid saliva) and 1 µl of saliva fluid were incubated in a buffer containing oligonucleotides-conjugated S1-RBD protein. Following incubation for one hour at 37 °C or overnight at 4 °C, PCR buffer was added, and then extension/prePCR and real-time PCR were performed. Saliva sample collected before the Covid pandemic was used as negative control. Delta Ct was calculated by subtracting Ct values of positive samples from those of negative controls. For a fair comparison, ∆Ct values of DSS were adjusted by adding 2 Ct to compensate for the fourfold greater amounts in the liquid samples.

Anti-S1-RBD antibody levels detected from DSS samples were slightly lower than those of wet saliva samples, except one sample whose ∆Ct was 1.15-fold lower in DSS compared to corresponding wet saliva samples (Supplementary Fig. S1). Comparisons between wet and dry saliva may be inaccurate due to pipetting errors handling small volumes of the variably viscous wet saliva. In addition, precipitates appeared when liquid saliva samples were frozen and thawed a few times. Our analyses showed that detection of antibodies using DSS samples generated consistent results (see below). Thus, DSS samples present advantages over wet saliva for detection of antibodies using AbPEA. While we have not been able to investigate the long term stability of antibodies in DSS, our previous study of DBS demonstrated that most proteins remained stable for decades in samples stored at + 4 °C^19^.

**Fig. 3:**
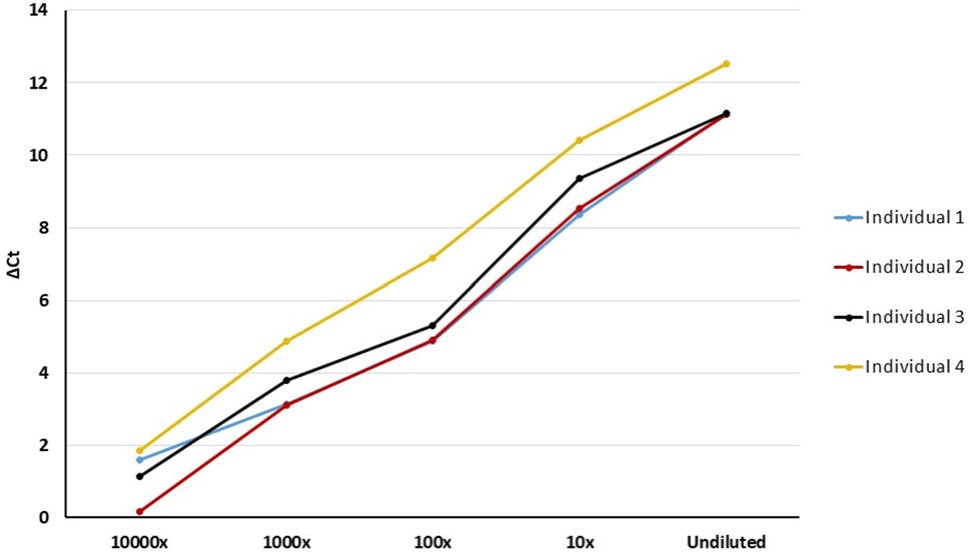
Sensitivity and dynamic ranges of anti-S1-RBD antibodies detection in DSS. DSS samples collected from 4 vaccinated individuals were tested. Paper discs, 1.2 mm in diameter (corresponding to approximately 0.25 µl fresh saliva liquid), were cut from a DSS and either added directly into the incubation solution (undiluted) or using 1 µl of s serial dilution, of up to 10,000-fold (corresponding to 1/4, 1/40, 1/400, 1/4000, 1/40,000 µl liquid saliva). Lines in four different colors represent values for four different individuals. ΔCt values were calculated using the Ct of dilution buffer minus that of Ct of sample at different concentration. The dynamic range spanned more than 3 logs and results of all investigated concentrations were within the linear range.

The sensitivity of AbPEA using DSS was tested through limiting dilution of samples eluted from DSS from four individuals (Fig. 3). Antibodies were detected directly from a cut from DSS samples (undiluted) or first eluted from one cut of ø1.2 mm disc in 10 µl elution buffer, and further ten-fold diluted. A ø1.2 mm disc or one microliter of each dilution was used which corresponds to 1/4, 1/40, 1/400, 1/4000 or 1/40,000 µl, respectively, of liquid saliva. ∆Ct values remained greater than 2 even after 1000-fold dilution for all individuals. Using the cut-off value of ∆Ct 2 defined in our previous study, all four samples were scored as positive even after 1000-fold dilution^4^. The dynamic range spanned more than 3 logs. Since the signals detected directly from one ø1.2 mm DSS disc without elution fell within the linear range, there was no need for elution and dilution. Consequently, detecting antibodies using DSS is more straightforward for monitoring SARS-CoV-2 antibody levels compared to DBS samples, which require dilution to achieve quantitation within the linear range.

The reproducibility of antibody detection was tested using 4 DSS samples, collected over four consecutive days (day 1, 3, 5 and 8) from 5 individuals. These DSS samples were kept at 4 °C or room temperature until tested. Very similar antibody levels were observed during this period with CV values for individual samples ranging from 2.54 to 5.35% for ∆Ct values of DSS kept at 4 °C and 4.36–9.68% for DSS stored at room temperature (Supplementary Fig. S2).

### Correlation of total anti-SARS-CoV-2 antibodies level between DSS and DBS

**Fig. 4:**
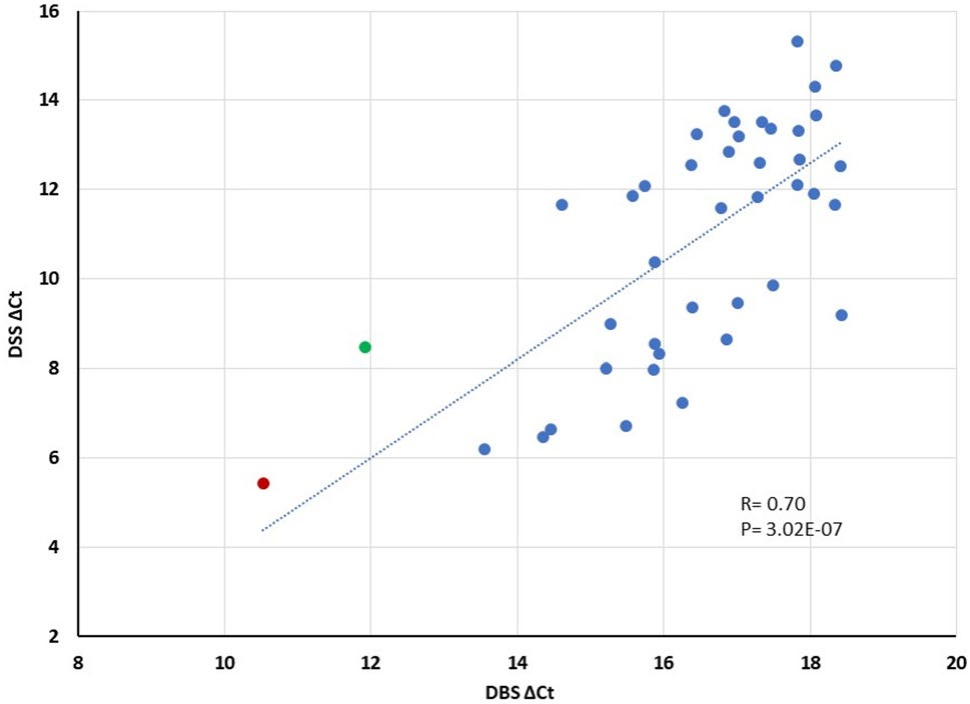
Correlation between anti-SARS-CoV-2 antibody levels in DSS and DBS. DSS and DBS were collected in parallel from 42 healthy individuals during the autumn of 2023. Total Ig was detected using a pair of oligonucleotides-conjugated S1-RBD. For DSS cuts of ø1.2 mm, corresponding to 0.25 µl saliva were used directly. For DBS cuts of ø1.2 mm from, corresponding to 0.5 µl fresh blood sample, were eluted and 1 µl of 100-fold dilutions were used to avoid signals at plateau levels. Two individuals had low antibody levels: one having only received two vaccinations (red dot) and another person (green dot) had only been infected once and was never vaccinated.

DSS and DBS were collected from 42 healthy individuals during the autumn of 2023 through unsupervised self-sampling. For the detection of antibody in DSS, ø1.2 mm cuts were directly incubated with a buffer containing oligonucleotide-antigen probes. However, for the DBS detection, ø1.2 mm cuts were incubated in 100 µl elution buffer to extract the antibody first, and 1 µl of elute (100-fold dilution) were used for the assay in order to achieve accurate quantification. All 42 DBS and DSS samples were analyzed in the same 96-well plate, together with a serial dilution of rabbit anti-S1-RBD antibodies using the same reaction conditions. To compensate for differences in the amounts of material from the different sample types (also taking into account that one ø1.2 mm DBS disc corresponds to 0.5 µl wet blood, and one ø1.2 mm DSS to 0.25 µl saliva liquid), 5.64 (log_2_50) was added to the final ∆Ct values for DBS, corresponding to the 50-fold less sample input from DBS over those in DSS. As shown in Fig. 4, all 42 DSS samples were positive and antibody levels ranged from ∆Ct of 5.5 (or 45-fold in ratio) up to 15.5 (46,000-fold) over the negative control (Y axis). Adjusted antibody levels in DBS ranged from ∆Ct 10.5 up to 18.5 (X axis). Antibody levels in DSS showed a greater variability among donors than those in DBS. Among two individuals with low levels of antibodies, one had received only two vaccinations (red dot) and another had undergone an infection but not received any vaccination (green dot). All other individuals had been vaccinated more than three times, with or without having also been infected. These clinical histories are consistent with the low recorded antibody levels in two of the individuals. Total antibody levels in DSS correlated well with those in DBS with a correlation coefficient of R = 0.70. Total antibody levels in DSS were generally 3–7 ∆Ct (corresponding to 10–100-fold) lower than those in DBS. These results are consistent with previously published differences of IgG levels between DBS and DSS samples, with R = 0.67^20^. Because of the convenient sample collection and no need for elution and dilution, DSS is an ideal sample for detection of specific antibody responses.

**Fig. 5:**
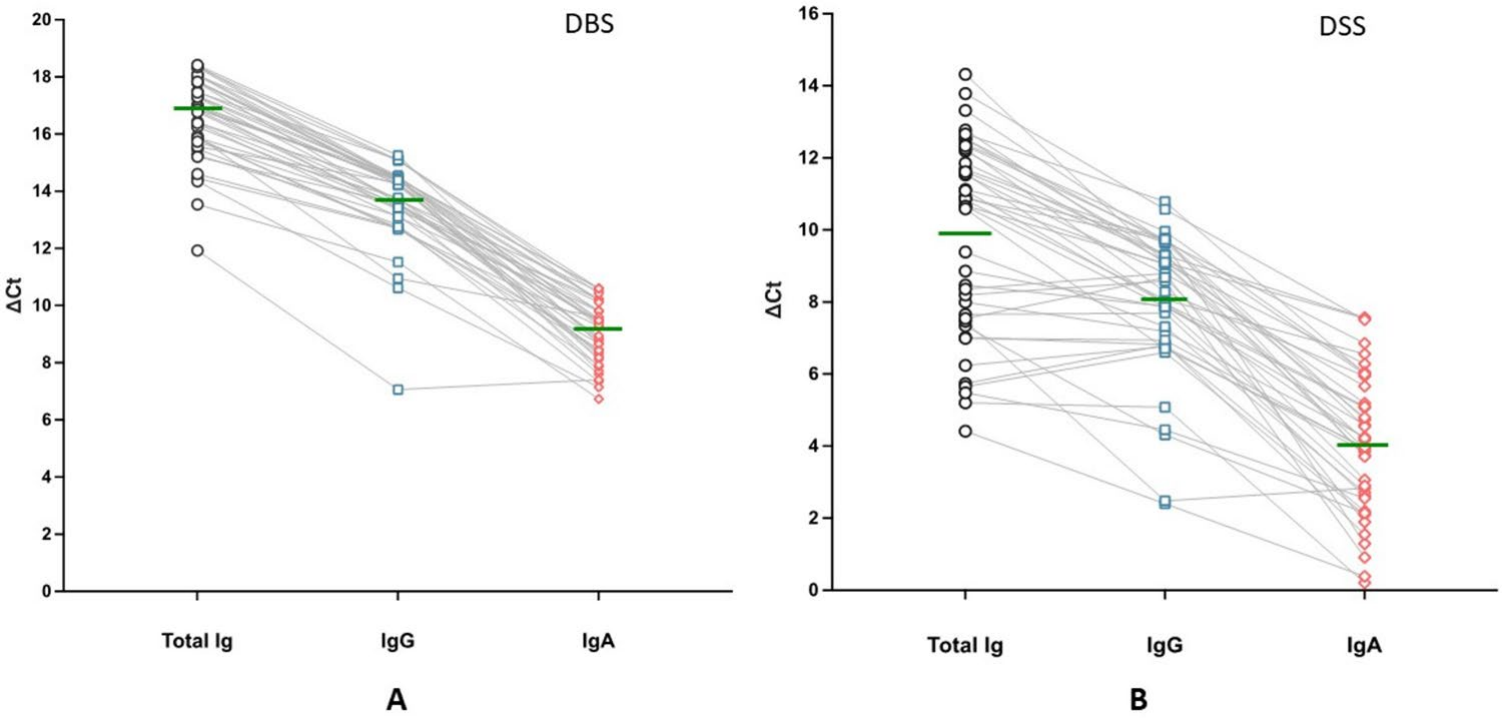
Detection of total Ig, IgG and IgA in DBS (**A**) and DSS (**B**) obtained from 42 individuals during the autumn of 2023. For the DBS detection, antibodies were eluted first from ø1.2 mm cuts in 100 µl elution buffer, and then 1 µl of elute (100-fold dilution) was used for the assay. Whereas the detection of antibody in DSS, ø1.2 mm cuts were directly incubated with a buffer containing oligonucleotide-antigen probes. All 42 DBS and DSS samples were analyzed in the same 96-well plate. To compensate for differences in the amounts of material from the different sample types 5.64 (log_2_50) was added to the final ∆Ct values for DBS, corresponding to the 50-fold less sample input from DBS over those in DSS. Lines in the middle of each group represent the average ∆Ct values. The corresponding of total and IgG and IgA levels for individual sample donors are indicated by grey lines.

We also analyzed total Ig, IgG, IgA and IgM reactivities to S1-RBD in DBS and DSS samples from the same 42 individuals as shown in Fig. 5A,B. The ∆Ct values for total antibodies in the 42 DBS samples ranged from 11.9 to 18.4, with an average ∆Ct of 16.6. The levels of IgG ranged from 7.1 to 15.3, with an average ∆Ct of 13.6 (Fig. 5A). IgA levels were relatively low as ∆Ct ranged from 6.7 to 10.6, and an average ∆Ct of 9.1. All ∆Ct of total Ig, IgG and IgA detection were more than ∆Ct of 6 (corresponding to 64-fold) above the cut-off value of ∆Ct of 2. Very similar correlation profiles between total Ig, IgG and IgA levels were observed for most individuals. Comparison of the correlations between IgG or IgA vs. total Ig or between IgG and IgA are shown in Supplementary Fig. S3. Although there were strong correlations between both IgG and IgA to total Ig (R = 0.88 and 0.75) the correlation between IgG and IgA was less strong at R = 0.64. In the case of antibody detection in DSS, the ∆Ct value for total antibodies spanned from 4.4 to 14.3, with an average ∆Ct of 11.8, whereas IgG varied from 2.4 to 10.8 with an average ∆Ct of 8.0 (Fig. 5B). IgA levels were relatively low as ∆Ct spanned from 0.2 to 7.5 with an average ∆Ct of 4.07. Six samples were below the cutoff value of ∆Ct of 2 described in our previous paper^4^. Correlation between total IgG and IgA levels were with R = 0.60, lower as compared to the correlations between IgG and total Ig, as well as between IgA and total Ig, both of which were R = 0.79 (Supplementary Fig. S4). Most IgG in saliva is derived from the microvasculature of gingival crevices, whereas most IgA is produced by plasma cells (PCs) in salivary glands, although some is locally produced^10^. The different origins of the Ig isotypes may account for the lower correlation between total antibody levels in blood and saliva, and between IgG and IgA in saliva. It has been shown that IgM directed against SARS-CoV-2 spike protein in saliva could be detected 14 days after the onset of symptoms, but levels were low^11^. As expected we observed no IgM reactivity to S1-RBD in DBS and DSS, since all samples were collected from healthy individuals with no recent history of Covid-19.

In conclusion, we have demonstrated the high sensitivity, reproducibility and broad dynamic ranges of measurement of antibody levels in DSS by AbPEA. Since SARS-CoV-2 antibody levels could be recorded within the linear range directly in ø1.2 mm cuts from DSS, no elution and dilution were needed, only addition of reagents and incubation. In yet unpublished studies we have extended the range of investigated antigen specificities in AbPEA reactions, demonstrating good correlation with standard assays (results not shown). The ease of sampling DSS provides advantages over DBS. We also adapted AbPEA for detection of Ig of different isotypes. The observed antibody and isotype kinetics to vaccination during a first and second vaccination accorded well with previous observations, validating the accuracy of the AbPEA isotype methodology.

## Materials and methods

### Antibodies and SARS-CoV-2 antigens

AffiniPure goat anti-human IgA, α chain specific (Code number 109-005-011), affiniPure goat antibodies directed against human IgM, Fc5µ (109-005-129) and against IgG Fcγ (109-005-098) were purchased from Jackson ImmunoResearch. Recombinant S1-RBD was produced in 293F cells by the Mammalian Protein Expression (MPE) Core Facility at the University of Gothenburg as described previously^4^.

### Protein-oligonucleotide conjugation

All oligonucleotides were synthesized by Integrated DNA Technologies (IDT) and the sequences of all oligonucleotides in this study have been published previously^21^. Those used herein are described in Supplementary Table S1. Oligonucleotides-conjugated S1-RBD, which was prepared as described previously remained stable for over 3 years and still used in this study^4^. In addition, goat anti-human IgG, IgM and IgA were conjugated with Oligo-Click-REV. The extension primers included uracil residues in place of some T residues as indicated in Supplementary Table S1. The molecular beacon included the fluorophore FAM, and as quencher DABSYL, at either end.

### Antibody proximity extension assay (AbPEA)

Discs of ø1.2 mm were cut with a Uni-coreTM micro puncher (Cytiva) from dry blood or saliva samples collected on paper. The cut paper discs were either added directly to 4 µl reactions solution containing 100 pM oligo-conjugated S1-RBD or 20 nM secondary antibody probes in phosphate-buffered saline (PBS), containing 5 mM EDTA, 100 µg/ml salmon sperm DNA (Sigma Aldrich), 0.1% BSA, 1 mM biotin, 100 nM goat IgG, 0.05% tween 20. For analyzing dilutions, paper discs were first eluted in 10 or 100 µl PBS containing 0.5% tween 20, 1% BSA and 1% Halt protease inhibitor cocktail (Thermo Scientific) on a shaker, set at 900 rpm for 60 min at RT^22^. One µl aliquots of eluted material or dilutions thereof were mixed with 3 µl reagent solution as described above. After incubation at 37 °C for 1 h or 4 °C overnight with similar results, 96 µl extension and pre-PCR solution containing 100 mM Tris–HCl pH 8.5, 50 mM KCl, 1.5 mM MgCl_2_, 0.75% TritonX-100, 0.2 mM dNTP, 1 µM forward and reverse primer and 1 unit Onetaq Hotstart DNA polymerase (New England lab) was added to each sample. The reactions were incubated at 50 °C for 20 min, followed by 5-min heat-activation at 95 °C and then 17 cycles prePCR of 95 °C for 30 s, 54 °C for 1 min, and 60 °C for 1 min using a primer pair common for all extension products. For the subsequent qPCR detection, 2.5 µl of the extension/pre-PCR products were transferred to a 96 or 384-well plate and combined with 7.5 µl qPCR mix to a final concentration of 1X PCR buffer (Invitrogen), 2.5 mM MgCl_2_, 0.2 mM of each dNTP, 1.6 µM ROX reference Dye (Invitrogen), 0.3 µM molecular beacon, 0.9 µM of the appropriate amplicon-specific primer pair, 0.1 U uracil *N*-glycosylase and 0.5 U recombinant Taq polymerase (Invitrogen). Quantitative real-time PCR was run with an initial incubation at 25 °C for 30 min, followed by incubation at 95 °C for 5 min, and then 30 cycles of 15 s at 95 °C, and 1 min at 60 °C. To control for variation between experiments and microtiter plates, a standard serial dilution of rabbit anti-S1-RBD IgG (from 10 nM to 1 pM), extension/PCR control^19^ as well as positive and negative donor controls were also included in each 96 well microtiter plate.

### Collection of DBS and DSS

DBS and DSS were collected from 42 healthy individuals by unsupervised self-sampling during the autumn of 2023. For DSS sample collection, subjects were asked to deposit saliva in a micro tube 10 min after rinsing mouth with water or without rinsing and the fluid was then pipetted onto Whatman paper (903 Protein Saver Card, Cytiva), and dried for 1 h at room temperature (RT). In addition, DBS samples were also collected before vaccination and at different time points after first and second vaccinations, as described previously^4^. A drop of blood of about 10–50 µl from a finger prick was spotted on paper and dried for more than 60 min at RT, before storage at + 4 °C or − 20 °C. Due to the differences in density, one circle of ø12 mm marked in Whatman paper adsorbs 50 µl blood or 25 µl saliva. Accordingly, one cut of ø1.2 mm disc corresponds to approximately 0.5 µl fresh blood or 0.25 µl fresh saliva, as estimated from comparison of antibody levels in wet samples. These volumes were confirmed by comparing anti-S1-RBD antibody levels in 1 µl fresh blood sample versus one ø1.2 mm disc cut from a DBS sample. Similarly, antibody levels in 1 µl fresh saliva liquid were compared to those of ø1.2 mm discs of DSS. Results showed that antibody levels in 1 µl fresh blood were about 1 ∆Ct higher than those in one ø1.2 mm disc from a DBS, while ∆Ct from saliva liquid was 2∆Ct higher than that of a ø1.2 mm disc cut from a DSS (data not shown). A negative saliva sample, pooled from human donors and collected before November 2019, was purchased from LEE Biosolutions (MO, USA) (Cat# 991-05-P-PreC).

The Ct values are the PCR cycle at which a fluorescence signal exceeds a threshold value. ΔCt values are calculated as the Ct of the negative control minus that of the sample. The correlations between total Ig, IgG and IgA in DBS and DSS were calculated using the ΔCt value and R and P values were calculated using the built-in Excel functions.

The investigation was approved by the Swedish Ethical Review Authority (Dnr 2020-06575 and 2021-03952) and conducted according to the Declaration of Helsinki. All sample donors signed an informed consent form.

## Supplementary Information

Supplementary Figure S1.

Supplementary Figure S2.

Supplementary Figure S3.

Supplementary Figure S4.

Supplementary Table S1.

Supplementary Legends.

## Data Availability

All data generated or analyzed during this study are included in this published article and its Supplementary Information files.

## Abbreviations

AbPEA: Antibody proximity extension assay
DSS: Dried saliva spot
DBS: Dried blood spot
S1-RBD: Spike protein subunit 1 with receptor-binding domain
Ct: Cycle threshold
∆Ct: Ct value of negative control minus Ct value of positive sample
PBS: Phosphate-buffered saline
