# Supplementary figures and images for: Monitoring SARS-CoV-2 IgA, IgM and IgG antibodies in dried blood and saliva samples using antibody proximity extension assays (AbPEA)
Source: Sci Rep. 2024 Sep 17;14:21655. doi: 10.1038/s41598-024-72453-5 (PMC11408710; doi:10.1038/s41598-024-72453-5)

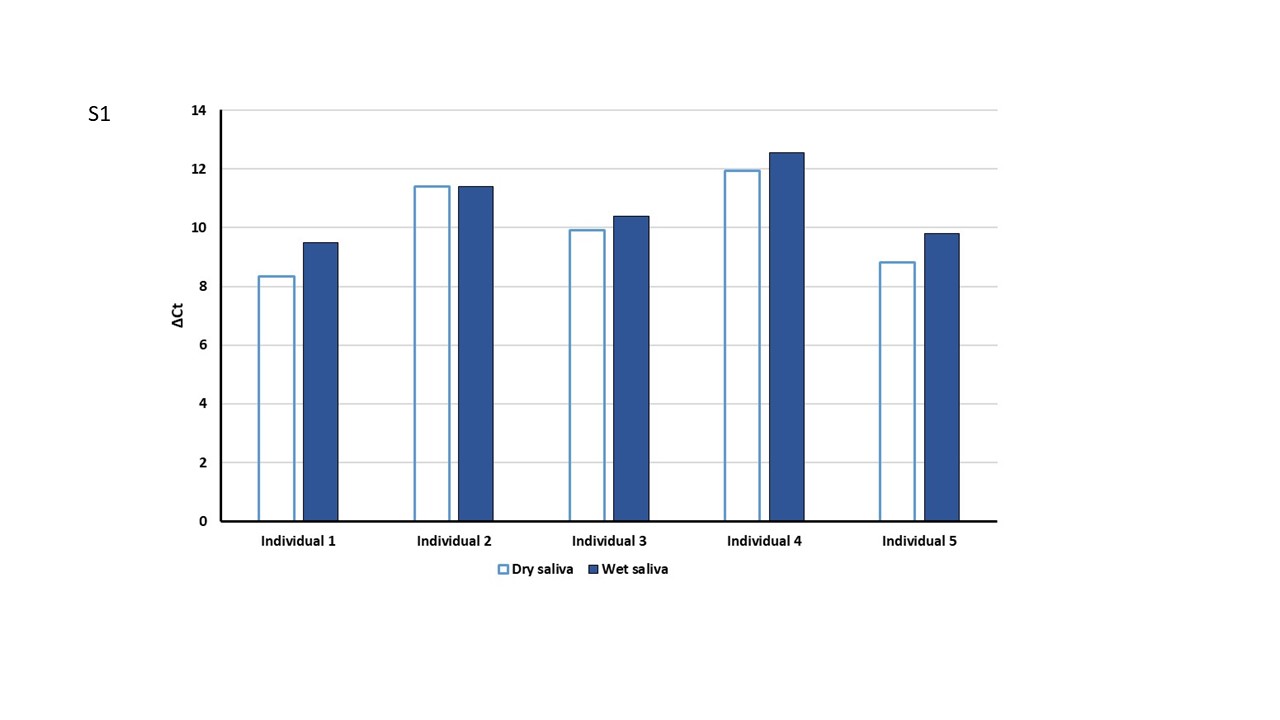

Supplement: Supplementary file 1 — Supplementary Figure S1. [file 41598_2024_72453_MOESM1_ESM.jpg]

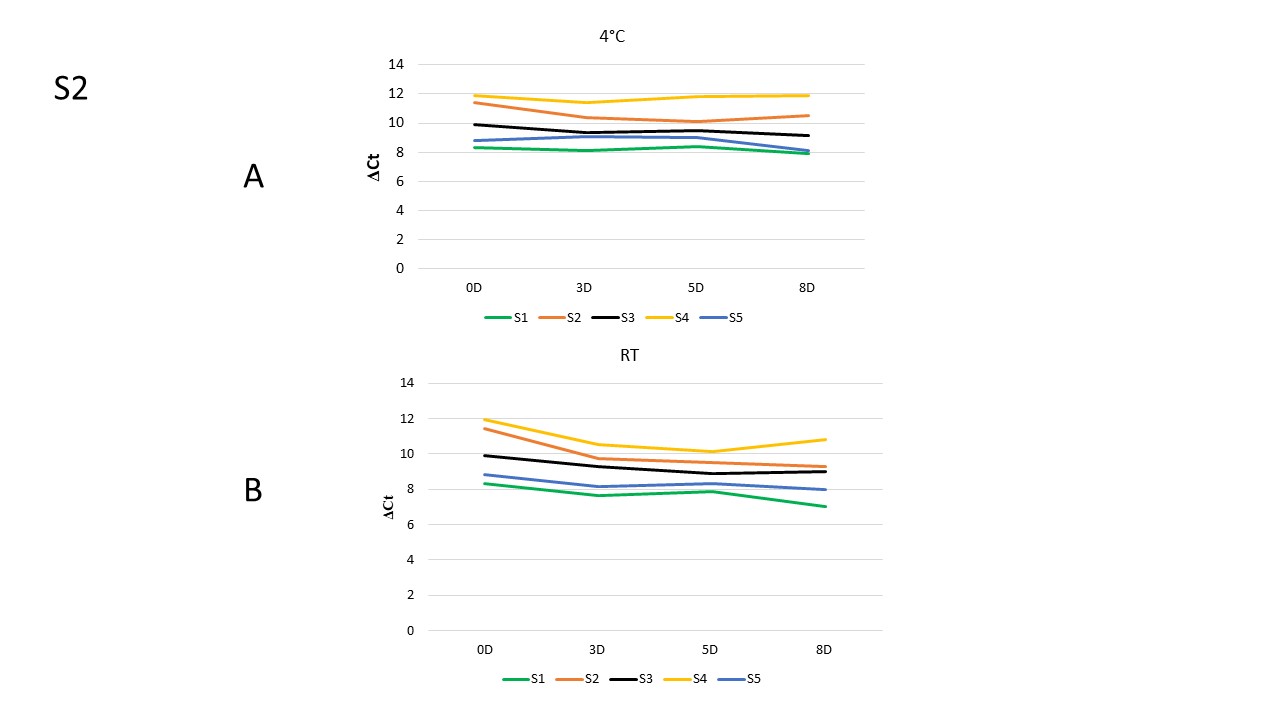

Supplement: Supplementary file 2 — Supplementary Figure S2. [file 41598_2024_72453_MOESM2_ESM.jpg]

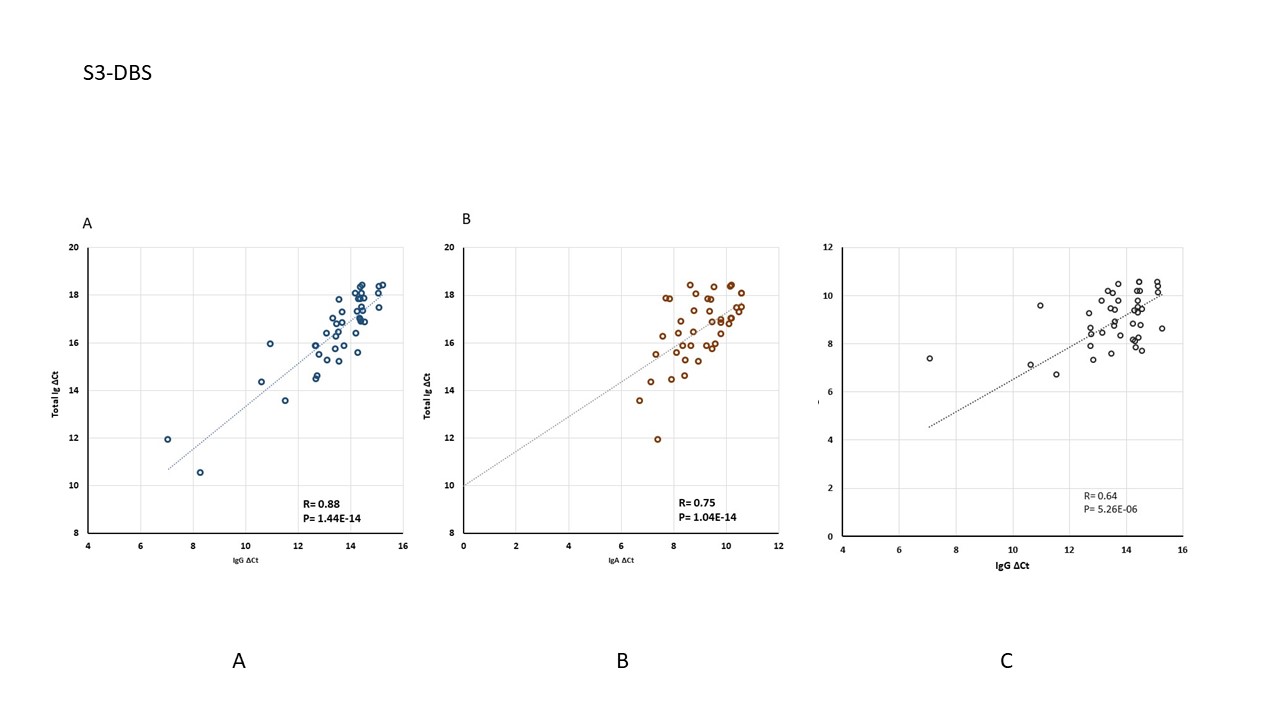

Supplement: Supplementary file 3 — Supplementary Figure S3. [file 41598_2024_72453_MOESM3_ESM.jpg]

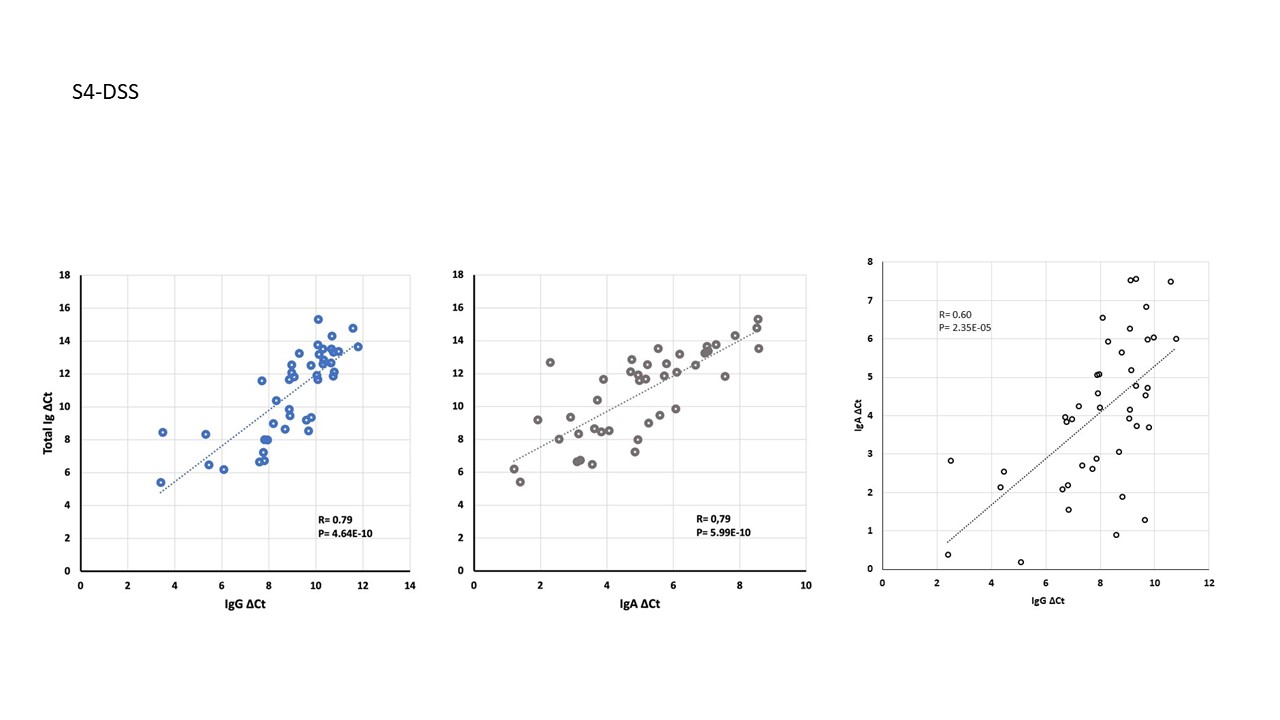

Supplement: Supplementary file 4 — Supplementary Figure S4. [file 41598_2024_72453_MOESM4_ESM.jpg]
