# Supplementary material for: Monitoring SARS-CoV-2 IgA, IgM and IgG antibodies in dried blood and saliva samples using antibody proximity extension assays (AbPEA)
Source: Sci Rep. 2024 Sep 17;14:21655. doi: 10.1038/s41598-024-72453-5 (PMC11408710; doi:10.1038/s41598-024-72453-5)
Supplement: Supplementary file 5 — Supplementary Table S1. [file 41598_2024_72453_MOESM5_ESM.docx]

| Protein/antibody | Oligonucleotides | Sequence (5'-3') |
| --- | --- | --- |
| S1-RBD | Oligo-Click-FWD1 | AzideN/CCACTGGGTCTGGTCAATCACGCCAGACGGTCAAATCCTCTAATCACGATGAGACTGGATGAA |
| S1, anti-IgG, M, A | Oligo-Click-REV | AzideN/ATATAGCTCGATTCCATGAACTTTCCCGTATAAACTC |
|  | Hyb-REV1 | GAGTTTATACGGGAAAGTTCATGGAATCGAGCCGTTGCCGTACTAGGGATACTTGCACCTTATGCTACCGTG  ACCTGCGAATCCAGTCT |
|  | Extenion-FWD primer | CCACTGGGuCTGGTCAAuCACG |
|  | Extesion-REV primer | GGGAAAGTuCATGGAAuCGAGC |
|  | Primer-FWD1 | CCAGACGGTCAAATCCTCTAA |
|  | Primer-REV1 | CGTTGCCGTACTAGGGATACT |
|  | molecular beacon | CCCGCTCGCTTATGCTACCGTGACCTGCGAATCCCGAGCGGG |
