# Supplementary material for: Monitoring SARS-CoV-2 IgA, IgM and IgG antibodies in dried blood and saliva samples using antibody proximity extension assays (AbPEA)
Source: Sci Rep. 2024 Sep 17;14:21655. doi: 10.1038/s41598-024-72453-5 (PMC11408710; doi:10.1038/s41598-024-72453-5)
Supplement: Supplementary file 6 — Supplementary Legends. [file 41598_2024_72453_MOESM6_ESM.docx]

**Supplementary Figure legends**

Figure S1. Comparison of antibody levels in dried and liquid saliva samples collected from five individuals. One cut of ø1.2 mm disc (equal to 0.25 µl liquid saliva) and 1 µl of saliva fluid were incubated in a buffer containing oligonucleotides-conjugated S1-RBD protein. A saliva sample collected in 2019 was used as negative control. The Y axis indicates the ∆Ct value calculated by subtracting Ct values of positive samples from Ct values of a negative control. Empty and solid columns display total antibody levels detected in DBS and liquid saliva, respectively.

Figure S2. Consistency of antibody detection in DSS collected from 5 individuals on four separate days; days 1, 3, 5 and 8 (indicated in 5 different color lines). DSS samples were stored at 4°C (A) or room temperature (B) before test. Cuts of ø1.2 mm disc from each DSS were incubated directly in a buffer containing oligonucleotides-conjugated S1-RBD protein. The ∆Ct values were calculated by subtracting Ct values of positive samples from that of a negative control.

Figure S3. Correlations between total levels of antibodies to S1-RBD vs. IgG antibodies (A) or vs. IgA antibodies (B) and between IgG vs. IgA antibodies (C) in DBS samples collected from 42 individuals. The same set of data used for Figure 5A was used in this figure. The correlation coefficients (R) are indicated in the figure.

Figure S4. Correlations between total Ig antibodies to S1-RBD vs. IgG (A) vs. to IgA (B), and correlation between IgG and IgA (C) in DSS samples collected from 42 individuals. The same set of data for Figure 5B was used in this figure. The correlation coefficients (R) are indicated in the figure.

Table S1 List of oligonucleotides used in this study

The oligonucleotide used for covalent coupling to antigen have an azide residue at their 5’ ends. S1/RBD were conjugated with Oligo-Click-FWD1 and Oligo-Click-REV. Goat anti-human IgG, IgM and IgA were conjugated with Oligo-Click-REV. The extension primers include uracil residues in place of some T residues. Primer-FWD1 and REV1 were used for real-time PCR. The molecular beacon includes a fluorophore, FAM, and a quencher, DABSYL, at either ends. Azide N stands for azide ester.
